# Optical Coherence Tomography Angiography Analysis of Retinal and Choroidal Vascular Networks during Acute, Relapsing, and Quiescent Stages of Macular Toxoplasma Retinochoroiditis

**DOI:** 10.1155/2020/4903735

**Published:** 2020-09-15

**Authors:** Georges Azar, Catherine Favard, Sawsen Salah, Antoine Brézin, Vivien Vasseur, Martine Mauget-Faÿsse

**Affiliations:** ^1^Eye & Ear Hospital International, Beirut, Lebanon; ^2^Faculty of Medicine, Saint Joseph University (USJ), Beirut, Lebanon; ^3^Faculty of Medicine, Holy Spirit University of Kaslik (USEK), Kaslik, Lebanon; ^4^Rothschild Ophthalmological Foundation, 25 Rue Manin, 75940 Paris Cedex 19, France; ^5^Université Paris Descartes, Centre d'ophtalmologie de l'Assistance Publique-Hôpitaux de Paris, Hôpital Cochin, 75014 Paris, France

## Abstract

**Purpose:**

To highlight the advantages of optical coherence tomography angiography (OCTA) in delineating the morphological features of the retinal and choroidal vascular network during acute, relapsing, and quiescent stages of macular toxoplasma retinochoroiditis.

**Methods:**

This prospective study included patients presenting with both active and quiescent ocular toxoplasmoses. OCTA was obtained to diagnose and follow the subsequent vascular network changes at diagnosis and six months after acute presentation.

**Results:**

Twenty-three eyes of 23 patients were included. In active lesions, OCTA showed extensive, well-delineated areas of intense hyposignal and perifoveal capillary arcade disruption in the parafoveal superficial capillary plexus (pSCP) and less extensive hyposignal in the parafoveal deep capillary plexus (pDCP). Signals of decreased deep capillary density and disorganization were also seen in the choroid. In nonactive lesions, OCTA demonstrated a homogenous and equally attenuated grayish hyposignal of the pSCP and pDCP and a partial restoration of the nonperfused choroidal areas.

**Conclusion:**

OCTA is a useful technique for vascular network analysis in toxoplasma retinochoroiditis. It allows the visualization of the different network changes and behaviors during the different stages of the infection.

## 1. Introduction

*Toxoplasma gondii* is a ubiquitous obligate intracellular parasite, which infects all warm-blooded vertebrates including humans as a zoonotic pathogen widespread in nature [1, 2]. *T. gondii* is transmitted by ingestion of undercooked meat containing toxoplasma cysts or tachyzoites, by contact with oocyte-infected feces, or through the placenta during pregnancy. More frequently, the pathogen is acquired postnatally, which also results in infection of neuronal tissues, and, in most cases, takes a clinically asymptomatic course [3–5]. Toxoplasma retinochoroiditis is a common form of “infectious” posterior uveitis. It accounts for 30 to 55% of posterior uveitis and is a leading cause of visual impairment [6, 7]. The classical clinical feature of ocular toxoplasmosis includes an exuberant vitritis with an area of active focal chorioretinitis. Occurring as a primary new focal area or more typically as a reactivation adjacent to an inactive choroidal scar, these lesions usually heal over several weeks to months and leave a quiescent, hyperpigmented chorioretinal scar [8, 9]. Optical coherence tomography angiography (OCTA) is a noninvasive diagnostic tool which allows visualization of retinal and choroidal structure using motion contrast. The generated vascular decorrelation signal enables visualization and theoretical quantification of the capillary density at different retinal and choroidal anatomical zones [10]. Although its indications in patient care are under continued clinical investigation, OCTA has shown promising results in demonstrating vascular changes occurring in common retinal pathologies such as choroidal neovascularization, diabetic retinopathy, and retinal vascular occlusions [11, 12]. However, this technique has not been used in analyzing retinal and choroidal vascular network behavior during the different stages of macular toxoplasma retinochoroiditis. Herein, we present a series of patients with clinical findings characteristic of ocular toxoplasmosis and highlight the advantages of OCTA in delineating the morphologic features of the retinal and choroidal vascular networks during acute, relapsing, and quiescent stages of macular toxoplasma retinochoroiditis. To the best of our knowledge, this is the largest ocular toxoplasmic series that studied with OCTA.

## 2. Materials and Methods

### 2.1. Baseline Data Collection

In this prospective study, twenty-three consecutive Caucasian patients (23 eyes) referred for retinal problems and diagnosed with both active and quiescent ocular toxoplasmosis were enrolled. Diagnoses were based on clinical characteristics consistent with macular toxoplasma retinochoroiditis, in the absence of other identifiable causes. Nineteen eyes were completely examined at the Odeon Center, Paris, France, whereas 4 eyes were initially examined at the uveitis department of Cochin Hospital and then referred for OCTA analysis at Odeon Center, between January 2015 and January 2018. All multimodal images including OCTA images were acquired and analyzed separately by two different retina specialists (C.F. at the Odeon Center and M.M.F. at the Rothschild Ophthalmological Foundation, Paris, France). Confirmatory serological testing was performed and showed high levels of IgM and IgG antibodies in 3 out of the 5 primary active cases and in 5 out of the 9 secondary recurrent cases. Information regarding age, sex, ethnicity, history of ophthalmological diseases or surgeries, and lens status was also collected. All patients underwent a complete ophthalmic examination including a best-corrected visual acuity (BCVA) test with Snellen eye charts, intraocular pressure measurement with Goldman applanation tonometry, anterior segment examination, and dilated fundus biomicroscopy. Standard fundus fluorescein angiography (FFA), indocyanine green angiography (ICGA), and spectral-domain optical coherence tomography (SD-OCT; both B-scan and “en-face”) were performed in all patients. Approval for data collection and analysis was obtained from the institutional review board of the Rothschild Foundation ethics committee. The study complied with the Health Insurance Probability and Accountability Act of 1996 and followed the tenets of the Declaration of Helsinki. Written consent was given and signed by the patients for their information to be stored in the hospital database and used for research prior to enrollment. All patients received a thorough explanation of the nature of the imaging modalities entailed in the study and the expected complications of FFA and ICG.

### 2.2. Image Acquisition and Analysis

Seventeen eyes were examined using OCT2 Spectralis Heidelberg Engineering, Germany (870 nm and 70000 A-scans/s), with software algorithm of OCTA Spectralis: Heyex software version 1.9.201.0, and 6 eyes were examined using the DRI OCT Triton swept source OCT, Topcon (1050 nm and 100000 A-scans/s) [11]. Three-dimensional 3 × 3 mm^2^ and 4.5 × 4.5 mm^2^ OCT “en-face” scans, centered on the fovea and coregistered with the cross-sectional OCT B-scans, allowed visualization of both retinal and choroidal flow and structure. Foveal thickness ((FT), defined as the average retinal thickness within the central 1 mm diameter ring) and subfoveal choroidal thickness ((SFCT), defined as the distance from the outer portion of the hyperreflective line (corresponding to the retinal pigment epithelium (RPE)) to the inner surface of the choroidal-scleral junction) were automatically carried out by enhanced depth imaging- (EDI-) OCT scans. Angiograms were generated using the built-in software to define the parafoveal superficial capillary plexus (pSCP) and the parafoveal deep capillary plexus (pDCP). Automated segmentations defined the pSCP from the internal limiting membrane to the boundary between the inner plexiform layer (IPL) and the inner nuclear layer (INL) and the pDCP from the boundary between IPL and INL to the boundary between the outer plexiform layer (OPL) and the outer nuclear layer (ONL). The choriocapillaries and choroid were analyzed using user-defined 80 *μ*m slabs distant from the basal membrane of -40 *μ*m for choriocapillaris and -118 *μ*m for the choroid. For the CC, the inner and outer boundaries were set at 31 *μ*m and 59 *μ*m beneath the RPE reference line, respectively. The RPE reference line is located at the middle of the hyperreflective RPE band on OCT. For all the cases, segmentation precision was checked and manual segmentation was then performed to correct device error. The algorithm identifies the voxels that belong to vessels and plots them by the ratio of inner and outer vessels. Outer voxels are neighbored nonvessel voxels. This ratio determines vessel size, and large vessels (large ratio between inner and outer voxels) located closer to the sclera are assigned to Haller's layer while vessels that are located above them and are smaller belong to Sattler's layer. All images were independently reviewed, and manual segmentation was performed by two retina specialists (C.F. and M.M.F.) until complete agreement was obtained between them. Observers were questioned about the absence or presence and location (whether in pSCP, pDCP, Haller's layer plexus, Sattler's layer plexus, or CC) of microvasculature changes.

### 2.3. Vessel Density Evaluation

The “parafoveal” area is defined as the annulus centered on the fovea with inner and outer ring diameters of 1 and 3 mm, respectively. With the current absence of blood vessel density analyzer software, only a descriptive analysis of toxoplasmic areas behavior was obtained comparing early phase angiograms and OCTA images at the level of the inner retina, outer retina, choriocapillaris, Sattler layer, and Haller layer. OCTA signals were described as intense hyporeflective, normal, or hyperreflective signals.

### 2.4. Treatment and Follow-Up

All patients in an acute phase were treated with oral antiparasitic medication (50-100 mg pyrimethamine and 250 or 500 mg azithromycin) along with anti-inflammatory medication (oral prednisone 60 mg/day) after 1 day of antiparasitic treatment. Oral steroids were then tapered over 1 month to 10 mg/d, then gradually tapered off while cotrimoxazole was discontinued after 2 weeks. In order to provide an optimal investigation about the vascular network behavior during the atrophic stages, OCTA imaging of all retinal and choroidal segmented layers was performed at 6 months after acute presentation.

### 2.5. Data Analysis and Statistical Methods

Clinical and imaging data were analyzed with frequency and descriptive statistics. Statistical analysis was performed using commercially available software SPSS (version 19.0, SPSS Inc., Chicago, Illinois, United States). A level of *P* < 0.05 was considered statistically significant, when applicable.

## 3. Results

### 3.1. Study Population

The study included 23 eyes of 23 patients (15 women and 8 men), with a mean age of 36 years (range 18-70; SD 10.6), diagnosed with macular toxoplasma retinochoroiditis. There were 5 cases (21.8%) of primary active area of retinochoroiditis, 9 cases (39.1%) of secondary satellite active lesion adjacent to an old retinochoroidal scar, and 9 cases (39.1%) of isolated quiescent scars with an atrophic, “punched-out” appearance and variable pigmentary changes. The profiles of the study subjects are listed in Table 1.

### 3.2. Image Analysis

#### 3.2.1. Active Lesion Analysis

**Figure 1:**
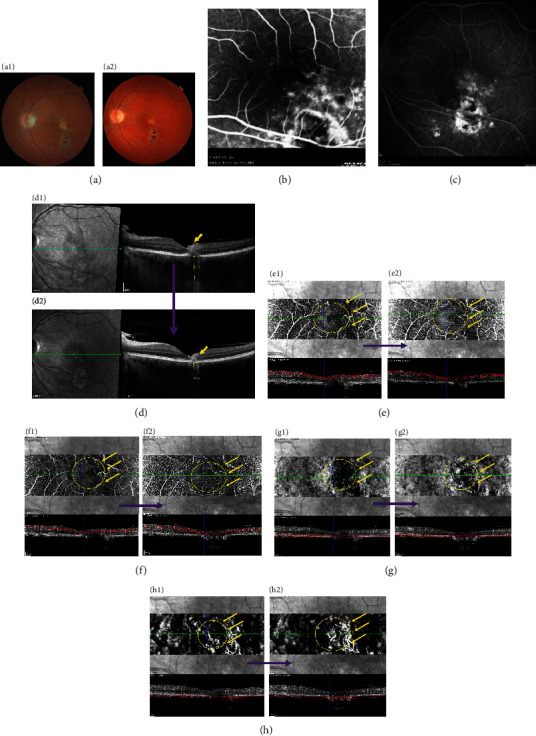
(a–h) Active recurrent toxoplasma retinochoroiditis and evolution after treatment, in a 30-year-old 6-month pregnant woman, presenting with an initial decreased vision from 20/20 to 20/100 and regaining to 20/63 after treatment with pyrimethamine, azithromycin, and 60 mg prednisone. (a) Shows an active yellowish retinitis lesion in the foveal region due to recurrence of toxoplasmosis (A1), with reduction of size of the relapsing lesion (A2) 1 month after antiparasitic and steroid treatment (color picture (Topcon, TRC)). Fundus fluorescein angiography (FFA) early (b) and late (c) phases show progressive hyperfluorescence with centripetal peripheral staining of the recurrent lesion (yellow arrows). Enhanced-depth imaging (EDI) optical coherence tomography (OCT) shows a pachychoroid with hyperreflectivity of all retinal layers of this retrofoveolar recurrent lesion (D1), with decreased size and thickness of the lesion 1 month after treatment (D2) (yellow arrows). Optical coherence tomography angiography of the active lesion shows areas of temporofoveolar capillary loss more extended in the parafoveal superficial capillary plexus ((pSCP), 50 *μ*m slab at +61 *μ*m/internal limiting membrane (ILM), E1) than in the parafoveal deep capillary plexus ((pDCP), 50 *μ*m slab at +108 *μ*m/ILM, F1). At the choroid level, OCTA shows areas of capillary and vessel loss less extended in the choriocapillary layer ((CC), 80 *μ*m slab at -40 *μ*m, G1) than in the deeper choroid (80 *μ*m slab at -118 *μ*m, H1). One month after treatment, OCTA shows the reappearance of some retinal capillaries at the level of the pSCP (E2) and the pDCP (F2). Other capillaries and larger vessels were also seen to have reappeared at the level of the CC (G2) and deeper in the choroid (H2).

In active lesions (Figure 1 A1–H1), FFA revealed early blockage from the area of the focal active retinitis or retinochoroiditis and from the chorioretinal pigmented scars in relapsing cases. A progressive centripetal hyperfluorescence of the active area with an adjacent well-circumscribed area of leakage apparent in the late frames was demonstrated in all patients (Figures 1 A1, 1(b), and 1(c)). SD-OCT (Figure 1 D1) demonstrated systematically increased retinal reflectivity and thickness of the active lesions. In all active toxoplasmic lesions, retinal hyperreflectivity was always progressing from the inner retinal layers to the external retinal and choroidal layers at the level of the active lesion. Moreover, outer retinal damage was always associated with inner retinal damage, which suggests that toxoplasmic lesions always progress from the inner retinal layers toward the outer retinal layers and choroid levels. Shadowing of the retinal pigment epithelium (RPE) and choriocapillaries was also noticed due to the inner retinal layer hyperreflectivity of the active lesion. Subretinal fluid (SRF) near the active lesion was seen in 6 patients (42.9%). These patients did not have any other associated findings elsewhere on the acquired images that differed than patients without SRF. In this active phase, there was an irregularly thickened hyperreflective interface appearing deep into the ONL lining the superior aspect of the subretinal fluid space around these focal active lesions. Those active inflammatory toxoplasmic lesions appear thick and hyperreflective on OCT. They have a masque effect on choroidal vessels, appear hypofluorescent on ICGA, and impair visualization of retinal and choroidal vessel signal on OCTA. Multiple hyperreflective dots could also be seen within the subretinal space as well as in the vitreous, inducing masque effects on ICG and on OCTA images.

As presented earlier, fundus vasculature was illustrated for all patients by OCTA at different retinal and choroidal layers (Figures 1): pSCP, pDCP, the CC, and Haller's layer. Among the 14 focal active lesions of necrotizing retinochoroiditis that included 5 primary active and 9 satellite lesions to adjacent atrophic scars, OCTA showed extensive, well-delineated, area of intense hyporeflective signal of non- or hypoperfusion and perifoveal capillary arcade disruption in the pSCP (Figure 1 E1) in all eyes (100%). In the pDCP, the area of hypoperfusion with capillary rarefaction appeared less extended than in the pSCP in all our cases (Figures 1 E1–F1). In the CC (Figure 1 G1) and Sattler layers (Figure 1 H1), capillary nonperfusion was observed in the same area as in the above retinal capillaries. All the hypoperfused lesions were confirmed on structural B-scan analysis to better appreciate the flow. Five cases could be analyzed with OCTA one month after combined antiparasitic and steroid treatment (Figures 1 A2–H2). In all these cases, OCTA showed, both in the pSCP (Figure 1 E2) and the pDCP (Figure 1 F2), that some capillaries seemed to reappear in the area where they had previously disappeared (yellow arrow). Moreover, some other thin capillaries reappeared in the area of capillary loss both in the CC layer (Figure 1 G2) and in the Sattler layer (along with other larger vessels (Figure 1 H2)).

One patient presented a choroidal new vessel defined as tiny hyperreflective vessels at the level of the RPE on OCTA, which was associated with the toxoplasmic lesion.

Finally, EDI-OCT showed that the mean choroidal thickness beneath the active lesions was 479.93 ± SD 103.26 *μ*m, which significantly decreased to a mean of 206.20 ± SD 18.80 *μ*m in the atrophic scars (*P* < 0.05, paired *t*-test).

Fourteen patients in the acute phase were treated with oral antiparasitic medication along with anti-inflammatory medication after 1 day of antiparasitic treatment. Six patients with macular lesion were treated by 3 days of 500 mg dexamethasone intravenous infusion initially. Then, oral steroids were tapered over 1 month to 10 mg/d, then gradually tapered off while cotrimoxazole was discontinued after 2 weeks. During follow-up, OCTA angiograms, which were done on the same previously active segmented lesions, revealed in all cases (100%) a partial restoration of the choroidal nonperfused areas in all layers (Haller's, Sattler's, and CC layers), as well as a partial decrease of the vascular network attenuation in both the pSCP and pDCP. Moreover, when treated earlier, OCTA showed that active toxoplasmic lesions could be stopped before reaching the outer retina or the choroid in 3 patients.

#### 3.2.2. Nonactive Lesion Analysis

**Figure 2:**
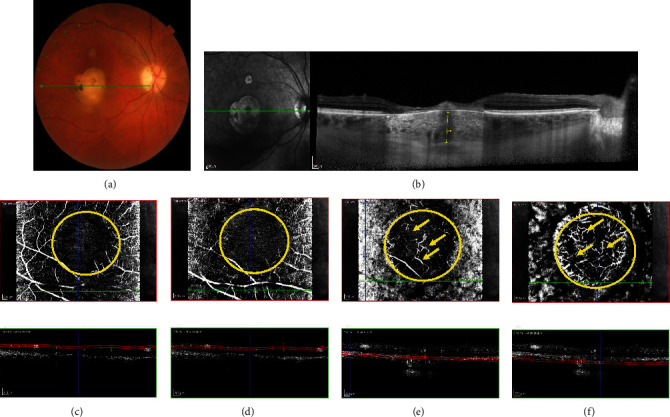
(a–f) Nonactive toxoplasmic atrophic scar in a 49-year-old woman with an inactive congenital toxoplasmic macular scar, with a best-corrected visual acuity (BCVA) of 20/100. Color retinophotography (a) shows a large atrophic macular scar with a peripheral superior satellite scar. Enhanced-depth imaging (EDI) optical coherence tomography (OCT) (b) shows disorganization and condensation of the retinal layers' reflectivity due to scar formation and atrophy, associated with an enhanced visualization of a submacular pachychoroid. Optical coherence tomography angiography (OCTA (c–f)) shows disappearance of retinal capillaries in the area of this atrophic retinal scar; but the extension of capillary loss appears to be wider in the parafoveal superficial capillary plexus (pSCP (c)) than the parafoveal deep capillary plexus (pDCP (d)). Moreover, a partial restoration of the previously nonperfused choroidal areas could be seen by reappearance of central thin capillaries at the level of the CC (e) and denser and larger vessels deeper in the choroid (f).

In nonactive atrophic scars (Figures 2), there was a late hyperfluorescence apparent within the scar linked to the atrophy (Figure 2(a)). EDI-OCT revealed a disorganization of the retinal layers' reflectivity due to scar formation and central irregular thickening of the RPE causing marked attenuation of the underlying choroid surrounded by a zone of RPE atrophy and increased choroidal visibility (Figure 2(b)). Furthermore, an interruption of the inner/outer segment (IS/OS) junction (also called the ellipsoid zone) was noticed in 6 out of the 18 atrophic lesions (33.3%).

To provide an optimal investigation about the retinal and choroidal vascular network behavior during the atrophic stages, OCTA imaging of all retinal and choroidal segmented layers (Figures 2(c)–2(f)) was performed at 6 months after acute presentation. In our 9 cases of toxoplasmic scar analysis, OCTA showed that both pSCP (Figure 2(c)) and pDCP (Figure 2(d)) capillaries have completely disappeared with a similar extension at the area of the atrophic scar. This finding was detected as homogenous but attenuated grayish capillary bed signal, the surface of which was similar in both the pSCP and pDCP. OCTA analysis of the CC (Figure 2(e)) and Sattler layers (Figure 2(f)) also shows a similar extension of capillary dropout. However, a partial restoration of the previously nonperfused choroidal areas was seen on OCTA angiograms, as demonstrated by the reappearance of some small capillaries in the CC and some denser and larger vessels in the Sattler layer (yellow arrows). Those findings were detected in 12 out of the 14 active lesions (85.7%) during follow-up. Unlike the difference detected between choriocapillaris and Sattler layers during the acute stages, the ischemic areas were similar during atrophic stages. Overall, the residual disorganized ischemic areas were larger in the pSCP and pDCP compared to the choroidal segmented layers.

**Figure 3:**
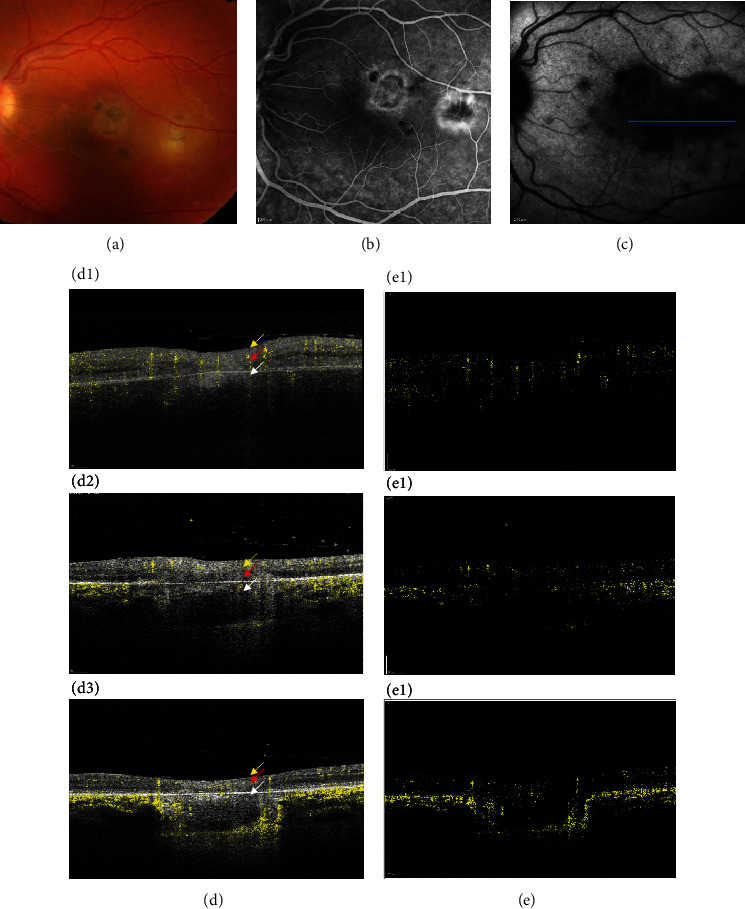
(a–e) Active recurrent toxoplasma retinochoroiditis in a 23-yo woman who presented with a best-corrected visual acuity (BCVA) of 20/40 in her left eye and treated with pyrimethamine, azithromycin, and prednisolone. (a) Shows a yellowish active temporomacular recurrence of a toxoplasmic lesion with centripetal hyperfluorescent leakage (color picture) at the late fundus fluorescein angiography (FFA) phase (b) and hypofluorescence of the whole lesion along with other hypofluorescent satellite spots on indocyanine green angiography (ICG) (c). (d) D1–D3 show the vessel flux evolution, respectively, at initial examination (D1), at 15 days after treatment (D2), and 2 months after treatment (D3), all in the superficial retinal capillaries (yellow arrows), deep retinal capillaries (red arrows), and choriocapillary vessels (white arrows). At presentation (D1), flux seems to disappear at the center of the toxoplasmic lesion site in all superficial and deep retinal capillaries and appears to be rarefied at the toxoplasmic lesion periphery. Also, vessel flux appears to be very weak and almost not detectable in the choroid. At day 15 (D2) and two months after treatment (D3), flux seems to reappear in some superficial and deep retinal capillaries, which may be due either to difficulties encountered during segmentation or the masque effect due to the inflammatory lesion, but either way seems to remain minimal. Finally, an increase of the choroidal flux can be observed at the periphery of the lesion after treatment. (e) E1–E3 correspond, respectively, to vessel flux shown in D1, D2, and D3, but with suppression of all retinal and choroidal layers.

The vessel flux evolution analysis between active lesions and atrophic scars is shown in Figures 3 D1–D3, which study the vessel flux evolution, respectively, at initial examination (Figure 3 D1), at 15 days after treatment (Figure 3 D2), and at 2 months after treatment (Figure 3 D3), all in the superficial retinal capillaries, deep retinal capillaries, and choriocapillary vessels. At presentation (Figure 3 D1), flux seems to disappear at the center of the toxoplasmic lesion site in all superficial and deep retinal capillaries and appears to be rarefied at the toxoplasmic lesion periphery. Also, vessel flux appears to be very weak and almost not detectable in the choroid. At day 15 (Figure 3 D2) and two months after treatment (Figure 3 D3), flux seems to reappear in some superficial and deep retinal capillaries, which may be due either to difficulties encountered during segmentation or to the masque effect due to the inflammatory lesion, but either way seems to remain minimal. Finally, an increase of the choroidal flux can be observed at the periphery of the lesion after treatment.

## 4. Discussion

Optical coherence tomography angiography (OCTA) is a novel noninvasive technology that can image retinal and choroidal microvascular structures based on detecting the level of motion between consecutive OCT images. Its noninvasive and 3-D nature allows frequent assessments of chorioretinal vasculature at various depths, with a precise anatomical location. There are few reports in the literature study in detail OCTA changes in retinal and choroidal vascular networks secondary to necrotizing toxoplasma retinochoroiditis. Besides, little data is available regarding those vascular networks' behavior during the different stages of infection [11–15]. In our study, we describe the first and largest cohort of patients presenting with toxoplasmosis and highlight the advantages of OCTA in delineating the morphologic features of the retinal and choroidal vascular networks during acute, relapsing, and quiescent stages of toxoplasma necrotizing retinochoroiditis. We also advocate some pathophysiological insights that may explain vascular network and flow behavior during the different stages of the disease [16].

In active lesions, OCTA showed extensive hypointense nonperfusion areas and perifoveal capillary arcade disruption in pSCP and fewer nonperfusion areas in the pDCP. Moreover, OCTA images through the choroid also showed a decrease of deep capillary density in Haller's layer and rarefaction of large linear vessels that were less prominent in Sattler's layer. A diffuse capillary network attenuation and disorganization were also seen in the CC. This vascular rarefaction, which could not be detected with FFA and conventional SD-OCT, is most probably due to a segmental retinal and choroidal periarteritis that leads to a local immunological response to toxoplasma, resulting in the deposition of immune cells and/or inflammatory debris within or adjacent to the vessel wall. Noteworthy, whether the vascular rarefaction is secondary to a local necrotic mechanism is yet to be verified.

On follow-up visits, all patients showing nonactive lesions exhibited partial and homogenous recovery of retinal and choroidal microvasculature, which was similar in both retinal (pSCP and pDCP) and choroidal (Haller's layer, Sattler's layer, and the CC) vascular networks. Although this vascular network behavior was predictable with resolution of the local inflammation next to chorioretinal nonactive scars, its pathogenesis remains highly debated, prompting us to further investigate competing theories [17, 18]. Initially, the dark hypointense CC areas seen on OCTA could either be due to a blockage of the flow signal by the clouded RPE and retinal layers' structure disruption due to active inflammation or to a real hypoperfusion of the CC. However, since the same hypointense areas were also seen in the superficial retinal layers, above which no shadowing-effect lesions were detected, we believe the hypointense nonperfusion areas are due to a real hypoperfusion of the CC rather than a blockage by the RPE edema or by the irregularly thickened hyperreflective interface appearing deep into the ONL around the focal active lesions.

Although OCTA images of most patients presenting with ocular toxoplasmosis studied were of high quality allowing visualization of the retinal and choroidal microvasculatures with high resolution and great details, limitations of our study have to be considered. First, the narrow field of view centered to the posterior pole limited our ability to delineate and cover all the extended peripheral areas where some microvascular retinal and choroidal network behavior could have been identified. Second, although measured objectively with a specific preset algorithm, the segmentation slab was not customized to adapt to the contour of the lesions (whether active lesions or atrophic pigmented scars), which may have interfered in the vascular network evaluation. Moreover, since OCTA segmentation of superficial and deep retinal capillary layers was difficult and associated with a masque effect due to the inflammatory active toxoplasmic lesion, it is difficult to ascertain that after healing, some capillaries may have reappeared. Third, only qualitative evaluation of the retinal and choroidal vascular networks was studied in different stages of toxoplasma retinochoroiditis areas. A more complete study design would have also included quantitative follow-up evaluation by calculating the adjusted flow index (AFI), defined as the average decorrelation value of all pixels above the noise threshold in the “en-face” angiogram and the percent area of nonperfusion (PAN), which corresponds to the percentage of pixels in each angiogram below the noise level [19, 20]. Those two parameters may be of great interest in assessing the vascular network evolution during inflammatory and atrophic stages. Besides, when studying the behavior of the vascular networks during the different stages of macular toxoplasmosis, some of our findings may be due to measurement bias. For instance, when we compared those networks before and after combined antiparasitic and steroid treatment, we noticed that in all these cases, OCTA showed, both in the pSCP and the pDCP, that some capillaries seemed to reappear in the area where they did not previously exist. Whether these capillaries were really absent during active stages or were unable to be detected by OCTA due to the edema or other imaging limitations is yet to be determined. Fourth, although larger than in previous investigations, the small numbers in our case series may preclude a generalized application of the result to all toxoplasma retinochoroiditis. Therefore, further investigations with larger prospective studies remain crucial to fully validate our results.

In conclusion, OCTA is a useful diagnostic tool for the noninvasive evaluation of retinal and choroidal vascular networks during acute, relapsing, and quiescent stages of retinochoroiditis caused by *Toxoplasma gondii*. Moreover, it can be a promising tool for clinicians to assess treatment efficacy in the follow-up visits, by evaluating restoration of the different vascular networks and visualizing the inversion of progression toward outer retinal and choroidal layers. Besides, it may assess the SFCT that seems to be thickened during active stages, and particularly recognize the origin of some hypofluorescent spots seen on FFA or ICG, which can correspond to blockage of the flow signal by the clouded RPE and deep retinal layer structure disruption due to active vitreoretinal inflammation.

## Figures and Tables

**Table 1:** Demographic characteristics of study eyes in cross-sectional comparison presenting with various stages of macular toxoplasma necrotizing retinochoroiditis.

	Primary active lesion	Secondary satellite active lesion	Atrophic scar
Number of eyes, *N* (%)	5 (21.8)	9 (39.1)	9 (39.1)
Mean age (standard deviation) (years)	35 (25)	31 (15)	40 (20)
Gender, male (%)	3 (60)	1 (11.1)	3 (33.3)
Snellen visual acuity	0.5	0.35	0.5
Mean subfoveal choroidal thickness (standard deviation) (*μ*m)	511 (176)	462 (116)	206.2 (18.8)

## Data Availability

The authors declare to have full access to the data analysis and take responsibility for the integrity and accuracy of the presented results.
